# Optimal surgical extent of lateral and central neck dissection for papillary thyroid carcinoma located in one lobe with clinical lateral lymph node metastasis

**DOI:** 10.1186/1477-7819-10-221

**Published:** 2012-10-25

**Authors:** Hyo Sub Keum, Yong Bae Ji, Jong Min Kim, Jin Hyeok Jeong, Woong Hwan Choi, You Hern Ahn, Kyung Tae

**Affiliations:** 1 Departments of Otolaryngology-Head and Neck Surgery, College of Medicine, Hanyang University, 222 Wangsimni-ro, Seongdong-gu, Seoul, 133-792, Korea; 2 Department of Internal Medicine, College of Medicine, Hanyang University, 222 Wangsimni-ro, Seongdong-gu, Seoul, 133-792, Korea

**Keywords:** Papillary thyroid carcinoma, Lateral neck dissection, Lymph node metastasis, Central neck dissection

## Abstract

**Background:**

The indications and extent of cervical lymph node dissection in papillary thyroid carcinoma (PTC) are still being debated. The aim of this study was to analyze the patterns of cervical lymph node metastasis in the lateral and central compartment and related factors and suggest the optimal extent of lateral and central neck dissection for PTC patients with clinical lateral lymph node metastasis.

**Methods:**

We retrospectively analyzed 72 patients with unilateral PTC who underwent therapeutic lateral neck dissections with concomitant total thyroidectomy and central neck dissection between January 2001 and December 2009.

**Results:**

The 72 patients underwent 79 sides of therapeutic lateral neck dissection. The most frequent metastatic level in the ipsilateral lateral compartment was level IV (75.0%), followed by level III (69.4%), level II (56.9%) and level V (20.8%). Multiple level metastases were common (77.8%) and were correlated with tumor size (≥ 10 mm). The central compartment lymph node metastasis rate was 87.5%, including 26.4% of contralateral central compartment metastases.

**Conclusion:**

In PTC patients with clinical lateral lymph node metastasis, the optimal extent of lateral and central neck dissection should include levels II, III, IV and V as well as the bilateral central compartment.

## Background

Papillary thyroid carcinoma (PTC) involves cervical lymph nodes in 30-80% of patients. Cervical lymph node metastasis in PTC has been found to be one of the most significant factors for locoregional recurrence, and it also has an adverse impact on survival, especially in older patients [1-3]. Therefore, appropriate treatment of overt and occult cervical lymph node metastasis acquires great importance for improving survival and minimizing regional tumor recurrence in PTC.

PTC with lateral compartment lymph node metastasis is considered more advanced than that with central compartment lymph node metastasis only. Dissection of the lateral compartments increases the frequency of complications because of the greater surgical invasiveness. Prophylactic lateral neck dissection is not generally recommended [4]. Because there is no evidence for the clinical significance of prophylactic lateral neck dissection, there is no consensus on its application among surgeons worldwide, although some Japanese surgeons recommend prophylactic lateral neck dissection for large primary tumor or massive extrathyroidal extension [5,6]. Therapeutic lateral neck dissection should be performed on patients who have PTC with clinically apparent cervical lymph node metastasis detected on palpation or imaging studies [7-9]. However, the extent of therapeutic lateral neck dissection remains controversial. Overall, there is no evidence to prove which approach is most appropriate for the management of lateral compartment lymph node metastasis, although most specialists believe that the berry-picking procedure should be avoided for initial therapeutic lateral neck dissection [10].

Therapeutic or prophylactic central neck dissection is usually performed simultaneously with therapeutic lateral neck dissection because of the high rate of central compartment lymph node metastasis. However, the extent of central neck dissection to be performed in clinically positive lateral compartment lymph node metastasis is also not clearly established. Some authors insist on ipsilateral central neck dissection, others on bilateral central neck dissection [11,12].

The aim of this study was to analyze patterns of lateral and central compartment lymph node metastasis and to suggest the optimal surgical extent of neck dissection in the lateral and central compartment for PTC patients with clinical lateral compartment lymph node metastasis.

## Methods

We retrospectively reviewed the clinical records of 72 patients with unilateral PTC who had undergone therapeutic lateral neck dissection with concomitant total thyroidectomy and central neck dissection between January 2001 and December 2009. All patients were confirmed as having lateral compartment lymph node metastasis by postoperative pathology reports. We excluded cases with bilateral PTC, recurrent cases or other types of thyroid cancer. We also excluded patients who had no pathologic lateral lymph node metastasis after therapeutic lateral neck dissection or had undergone neck dissection for other diseases. Written informed consent was obtained from the patient for publication of this report and any accompanying images. The Institutional Review Board of Hanyang University Hospital approved the study.

Physical examination, thyroid ultrasonography (US), neck computed tomography (CT) and fine-needle aspiration cytology were performed for preoperative evaluation of the primary thyroid tumors and suspicious cervical lymph nodes in the lateral and central compartment. The criteria for metastases requiring US were as follows: round shape (long/short ratio < 2), microcalcification, cystic change, hyperechogenicity and heterogeneous inner structure [13]. The criteria for CT were as follows: enhancement, heterogeneous, cystic or necrotic change and round shape. The size criteria for both US and CT were based on an upper limit of 15 mm for the nodal diameter of the normal long axis in the cases of jugulodiagastric and submandibular nodes and 10 mm for all other cervical nodes except for level VI [14]. We did not apply size criteria in the central compartment [15]. Neck US and serum thyroglobulin measurement (TSH-stimulated or TSH-suppressed thyroglobulin) were performed at 6- to 12-month intervals to detect recurrence. Mean follow-up period was 46.6 ± 31.9 months (range 11–118).

We did not perform prophylactic lateral neck dissection for PTC patients with clinically negative lateral neck. Levels II to V were routinely dissected in the lateral compartment. Level I was dissected only if there were radiographic, cytopathologic, or intraoperative findings suggestive of metastatic cancer. The dissected lateral compartment lymph node specimens were classified according to neck nodal level (levels I, II, III, IV and V). The central compartment lymph node specimens were also labeled by the surgeon according to their locations using the definitions of the central compartment and its subgroups in the recent consensus statement of the American Thyroid Association (ATA), and they were sent for permanent biopsy. The ipsilateral central compartment lymph node was defined as including pretracheal, prelaryngeal, ipsilateral paratracheal lymph nodes, and the contralateral central compartment lymph node included the contralateral paratracheal lymph nodes.

SPSS version 15.0 software (SPSS, Inc.) was used for statistical analysis. Fisher’s exact test was used to analyze the relationship between various clinicopathologic factors and cervical lymph node metastasis and recurrence (univariate analysis). Binary logistic regression analysis was used for multivariate analysis. A *P*-value of less than 0.05 was taken as statistically significant.

## Results

The characteristics of patients and tumors are listed in Table 1. The study group comprised 51 women (70.8%) and 21 men (29.2%). Mean age was 43.8 ± 14.2 years (range 15–80 years).

**Table 1:** **Patient demographics and tumor characteristics (*n*** **= 72)**

**Characteristics**	**Number of patients (%)**
Gender	Male:female = 21 (29.2%):51 (70.8%)
Age	43.8 ± 14.2 years (range 15–80 years)
Size	21.8 ± 13.1 mm (range 4–65 mm)
Location of tumor	
Upper third	18 (25%)
Lower two-thirds	54 (75%)
Multifocality of tumor	23 (31/9%)
Minimal extrathyroidal extension	50 (69.4%)
Maximal extrathyroidal extension	11 (15.3%)
Lymphovascular invasion	37 (50.7%)
T classification	
T1	14 (19.4%)
T2	7 (9.7%)
T3	46 (63.9%)
T4	5 (6.9%)
N classification	
N1b	72 (100%)
TNM stage	
Stage I	38 (52.8%)
Stage IVA	34 (47.2%)

Of 72 patients, a total of 79 sides of therapeutic lateral neck dissections were performed. Therefore, ipsilateral lateral neck dissection was performed in all patients and contralateral lateral neck dissection in seven patients (9.7%). All patients underwent lateral neck dissection including levels II-V in the neck each. There was one patient with suspicious metastatic lymph nodes at ipsilateral level I, so modified radical neck dissection including levels I to V was performed in this one (1.4%) patient.

All patients underwent therapeutic (32 cases, 44.4%) or prophylactic (40 cases, 55.6%) bilateral central neck dissection simultaneously with the lateral neck dissection based on imaging findings.

Among the 72 ipsilateral lateral necks, 75.0% of the lymph node metastases were in level IV, 69.4% in level III, 56.9% in level II and 20.8% in level V. There was no metastasis in level I in the final pathologic report. In the seven clinical contralateral lateral necks, the frequency of lymph node metastases was five in levels IV and III and three in level II.

The pattern of lateral compartment lymph node metastasis was not significantly different according to primary tumor location (upper third versus lower two-thirds of the thyroid gland).

Multiple level lymph node metastases in the lateral compartment were found in 56 (77.8%) patients (6 levels in 1 case, 5 levels in 2 cases, 4 levels in 6 cases, 3 levels in 22 cases and 2 levels in 25 cases). Single level lateral compartment lymph node metastasis occurred in 16 (22.2%) ipsilateral necks (9 cases in level IV, 4 cases in level III and 3 cases in level II). The multiple level lymph node metastases were associated with tumor size (≥10 mm) in multivariate analysis (*p* = 0.049) (Table 2). Seven patients who underwent therapeutic bilateral lateral neck dissection had lymph node metastasis in both sides of lateral compartment. No patients had only contralateral lateral compartment lymph node metastases. Six of the seven patients with contralateral lateral compartment lymph node metastases also had central compartment lymph node metastases, including five with bilateral central compartment lymph node metastases and one with ipsilateral central compartment metastasis. The occurrence of clinical contralateral lateral compartment lymph node metastasis was significantly associated with contralateral central compartment lymph node metastasis (*p* = 0.002).

**Table 2:** Statistical analysis of factors the related to multiple level lateral compartment lymph node metastases

	***P-value***	
**Factors**	**Univariate analysis**	**Multivariate analysis**
Gender	0.238	0.299
Age (45≤)	0.461	0.704
Stage	0.489	0.752
Multiplicity	0.363	0.050
Location of primary tumor (upper third or lower two-thirds)	0.638	0.670
Size of primary tumor	0.047	0.049
Minimal extrathyroidal extension	0.483	0.288
Maximal extrathyroidal extension	0.203	0.052
Lymphovascular invasion	0.276	0.065
Central neck metastasis	0.317	0.391
No. of metastatic nodes at level VI (≥2)	0.155	0.190

Central compartment (level VI) lymph node metastasis occurred in 63 (87.5%) (bilateral 18 cases, unilateral 45 cases) patients. Contralateral central compartment lymph node metastasis occurred in 19 (26.4%). One patient had only contralateral central compartment lymph node metastasis without ipsilateral central compartment metastasis. In multivariate analysis, contralateral central compartment lymph node metastasis was significantly associated with ipsilateral central neck metastasis and two or more metastatic central compartment lymph nodes (Table 3). Skip metastasis, defined as lateral compartment lymph node metastasis without central compartment lymph node metastasis, was found in eight (11.1%) patients. Skip metastasis was not correlated with any clinicopathologic factor.

**Table 3:** Statistical analysis of factors related to contralateral central compartment lymph node metastasis

	***P-value***	
**Factors**	**Univariate analysis**	**Multivariate analysis**
Gender	0.363	0.525
Age (45≤)	0.563	0.908
Stage	0.386	0.568
Multiplicity	0.124	0.148
Location of primary tumor (upper third or lower two-thirds)	0.575	0.873
Size of primary tumor	0.198	0.260
Minimal extrathyroidal extension	0.532	0.844
Maximal extrathyroidal extension	0.116	0.064
Lymphovascular invasion	0.107	0.129
Multiple level metastasis (≥2)	0.560	0.882
Ipsilateral central neck metastasis	0.035	0.038
No. of metastatic nodes in the ipsilateral central compartment (≥2)	0.031	0.017

The mean number of lymph nodes removed was 27.8 ± 14.39 (range 6–71) in the lateral compartment (8.53 ± 6.92, 7.29 ± 4.45, 8.22 ± 5.1 and 4.18 ± 4.31 in levels II, III, IV and V, respectively) and 7.91 ± 6.11 (range 1–35) in the central compartment.

Of the occult metastases in the ipsilateral lateral compartment, 42.2%, 47.3%, 64.5% and 23.6% were in levels II, III, IV and V, respectively. The sensitivities of the combination of US and CT for the detection of lateral cervical lymph node metastasis were 74.1%, 82.4%, 70.7% and 29.4% in levels II, III, IV and V, respectively. In the central compartment, the occult metastasis rate was 77.8% on the ipsilateral side and 27.0% on the contralateral side. The sensitivity of the combination of US and CT was 51.7% in the ipsilateral central compartment and 19.0% in the contralateral central compartment.

Recurrence occurred in seven (9.7%) patients: regional recurrence in four, locoregional recurrence in two and distant metastasis in one. Of the six patients with regional recurrence, four had recurrence in the dissected level (3 cases in level IV, 2 cases in level VI and 1 case in level V) and two in the undissected contralateral lateral neck. Recurrence was associated with stage in multivariate analysis (*p* = 0.032).

## Discussion

The pattern of lymphatic drainage of the thyroid is relatively consistent, and the spread of lymph node metastasis in PTC takes place in a stepwise fashion. Generally, cervical lymph node metastasis in PTC involves the central compartment first, followed by the ipsilateral lateral compartment and then the contralateral lateral compartment and mediastinal lymph node [16,17]. However, sometimes skip metastasis to the lateral compartment, bypassing the central compartment, has been reported [18]. The results of this study showed the similar lymphatic drainage pattern of PTC; most of the patients with lateral compartment metastasis also had central compartment metastasis, and patients with contralateral lateral compartment metastasis also had ipsilateral lateral compartment and contralateral central compartment metastasis.

Although several authors have described the frequency and pattern of lateral compartment lymph node metastasis, the optimal extent of therapeutic lateral neck dissection for PTC remains unclear. The ATA guidelines advocate compartment-oriented en bloc lateral neck dissection in patients with clinical lateral compartment lymph node metastasis, but offer no recommendation concerning which nodal levels should be dissected [4].

A formal modified radical neck dissection including levels I, II, III, IV and V is usually not necessary in PTC patients with lateral compartment lymph node metastases because of the great rarity of metastatic nodes at level I. We performed level I dissection only in one case with clinically apparent lymph nodes in level I, and the final pathology revealed no metastasis in level I in this study.

Several authors have reported that PTC metastasis is generally present at levels II through V in lateral compartment neck metastasis, with level III or IV nodes consistently the most frequently involved, and comprehensive neck dissection including levels II to V is necessary for complete clearance of lateral neck metastasis [19-21]. However, there is a question of whether or not to routinely dissect level V, considering the potential morbidity associated with injury to the spinal accessory nerve. Some authors raise objections to routine level V dissection for PTC patients with lateral compartment lymph node metastasis [22,23].

However, level V was found to be involved in a substantial proportion of cases in this study and previous studies [19,20]. In addition, preoperative imaging including US and CT was found to lack sensitivity for detecting metastatic disease in level V [24]. In this study, there was 20.8% level V metastasis on the ipsilateral side. The sensitivity of the combination of US and CT in level V was low (29.4%), and the occult lymph node metastasis rate of level V was relatively high (23.6%). Usually level V metastases are not found in isolation and instead are always associated with disease at multiple levels [19]. In this study, there was no case of single level V metastasis, and metastasis to level V was associated with level III metastasis in multivariate analysis (Table 4).

**Table 4:** Statistical analysis of factors related to level V metastasis

	***P-value***	
**Factors**	**Univariate analysis**	**Multivariate analysis**
Gender	0.720	0.568
Age (45≤)	0.744	0.529
Stage	1.0	0.150
Multiplicity	1.0	0.718
Location of primary tumor (upper third or lower two-thirds)	0.450	0.344
Size of primary tumor	0.197	0.150
Minimal extrathyroidal extension	1.0	0.817
Maximal extrathyroidal extension	0.672	0.771
Lymphovascular invasion	0.338	0.279
Central neck metastasis	0.337	0.173
Multiple level metastasis (≥2)	0.108	0.054
No. of metastatic nodes in the lateral compartment (≥5)	0.097	0.057
Contralateral lateral neck metastasis	1.0	0.845
Level II metastasis	1.0	0.861
Level III metastasis	0.048	0.043
Level IV metastasis	0.484	0.288
Level II or III metastasis	0.439	0.297
Level III or IV metastasis	0.337	0.173
Level II, III and IV metastasis	0.279	0.197

Given the evidence both for and against dissection of level V in PTC, we suggest, based on our results, that routine level V dissection is necessary, especially in PTC with level III metastasis, because of the frequent multilevel metastasis and the difficulty detecting level V lymph node metastasis preoperatively by imaging studies. When including level V dissection in lateral neck dissection, it is sensible to incorporate all areas of level V because there is no clear anatomical boundary between levels VA and VB, although some authors insist on elective dissection of only level VB, showing their results that metastatic PTC was identified in 0% of VA and 40% of VB [25]. In this study, we routinely dissected level VA and VB when performing level V dissection; however, we did not divide the surgical specimens into levels VA ad VB. Level VA dissection can be done with low morbidity and little additional time. There was no injury of the spinal accessory nerve or severe shoulder syndrome among patients who underwent level V dissection including levels VA and VB in this study.

In addition to controversy about level V dissection, some authors have also raised objections to routine level IIB dissection, to minimize damage to the spinal accessory nerve [25-27]. They recommended elective dissection of level IIB only when IIA is involved in the disease, because level IIB metastasis is usually accompanied by level IIA metastasis. In this study, we dissected level IIA and IIB together. However, we did not divide the level II specimens into level IIA and IIB in the most of the patients. Hence, we cannot throw any light on the issue of level IIB dissection. Further study involving separate sampling of level IIB lymph nodes is needed.

The central compartment lymph node is the most common site of metastasis in PTC, and most patients with lateral compartment lymph node metastasis also have central compartment lymph node metastasis [11,12,18]. Therefore, central neck dissection should be routinely performed during the initial operation when lateral compartment lymph node metastasis is suspected, regardless of whether central lymph node metastasis has been detected. However, the extent of the central neck dissection that should be performed in clinically positive lateral compartment lymph node metastasis has not been clearly established. Roh et al. recommended ipsilateral central neck dissection along with lateral neck dissection because of the low rate of contralateral central compartment lymph node metastasis (8.9%) [11]. However, Koo et al. insisted on bilateral central neck dissection due to the relatively high rate of occult contralateral central lymph node metastasis (34.3%), especially in multifocal primary tumors and when there is positive lymph node involvement at all lateral neck levels [12]. In the present study, the central compartment lymph node metastasis rate was very high (87.5%), and the contralateral central compartment lymph node metastasis rate was also relatively high (26.4%). Contralateral central compartment lymph node metastasis was significantly correlated with ipsilateral central compartment metastasis. The sensitivity of the combination of US and CT was very low (19.0%) in the contralateral central compartment, and the rate of occult metastasis in the contralateral central compartment was also relatively high (27.0%). Based on the results of this study, we recommend bilateral central neck dissection when therapeutic lateral neck dissection is performed because of the high rate of occult metastasis and the low sensitivity of imaging studies in the contralateral central compartment.

In this study, of six cases with regional recurrences, four occurred in the previously dissected level and only two occurred in an undissected contralateral lateral neck. Recurrence was more frequent in previously dissected areas than in those that had not yet been dissected, indicating that surgeons should take great care to achieve complete resection to prevent recurrence in the same area. The prognostic significance of persistence of occult micrometastasis or recurrence in cervical lymph nodes in PTC is still unknown. However, it might be clear that the complete removal of cervical lymph node metastases can prevent lymph node recurrence and reoperation of the recurrence.

## Conclusion

In PTC patients with clinical lateral lymph node metastasis, multiple level metastases in lateral compartment and concomitant central compartment lymph node metastasis were common. The optimal extent of lateral and central neck dissection should include levels II, III, IV and V as well as the bilateral central compartment.

## Abbreviations

PTC: Papillary thyroid carcinoma; ATA: American Thyroid Association; US: Ultrasonography; CT: Computed tomography.

## Competing interests

The authors declare that they have no competing interests.

## Authors’ contributions

HSK participated in the study design and data analysis and drafted the manuscript. YBJ and JMK carried out the data collection and performed the statistical analysis. JHJ, WHC and YHA provided the study material and reviewed the manuscript. KT participated in the study design and the interpretation of data and wrote the manuscript. All authors read and approved the final manuscript.
